# Clinical and epidemiological characteristics of whooping cough in hospitalized patients of a tertiary care hospital in Peru

**DOI:** 10.5935/0103-507X.20190029

**Published:** 2019

**Authors:** Pamela Yesquen, Eder Herrera-Perez, Raffo Escalante-Kanashiro

**Affiliations:** 1 Instituto Nacional de Salud del Niño - Lima, Perú.; 2 Universidad Peruana de Ciencias Aplicadas - Lima, Perú.; 3 Universidad Nacional Federico Villarreal - Lima, Perú.

**Keywords:** Pertussis/epidemiology, Whooping cough, Bordetella pertussis, Tos ferina/epidemiología, Tos convulsiva, Bordetella pertussis

## Abstract

**Objective:**

Describe the clinical and epidemiological characteristics of patients under 2 years of age hospitalized with whooping cough in a tertiary care children's hospital in Peru.

**Methods:**

This was a case series of patients under 2 years of age who were hospitalized with a diagnosis of whooping cough in 2012.

**Results:**

A total of 121 patients were hospitalized. Diagnostic testing (direct immunofluorescence, polymerase chain reaction, culture) was carried out in 53.72% of patients. Overall, 23.15% (n = 28) were confirmed cases, all of whom were patients less than 10 months old, and none of whom had received 3 doses of whooping cough vaccine. A total of 96.43% (n = 27) of cases were under 6 months of age, 42.86% (n = 12) were younger than 3 months, and 10.71% (n = 3) were admitted to the intensive care unit. Of these cases, all were younger than 2 months old, and one patient died. The most common symptoms in the confirmed cases were coughing (96.43%), facial redness (96.43%), paroxysmal coughing (92.86%), and coughing-related cyanosis (78.57%). The most frequent probable epidemiological contact was the mother (17.86%), and the majority of cases occurred in the summer (46.43%).

**Conclusion:**

Whooping cough is a cause of morbidity and mortality, especially in those younger than 6 months of age and in those who are not immunized or only partially immunized. Vaccination rates should be improved and case confirmation encouraged to prevent the underdiagnosis of this disease.

## Abstract (es)

**Objetivo:**

Describir las características clínicas y epidemiológicas de los pacientes menores de 2 años hospitalizados con el diagnóstico de tos ferina en un hospital pediátrico de tercer nivel de Perú.

**Métodos:**

Serie de casos de pacientes menores de 2 años hospitalizados con diagnóstico de tos ferina durante el año 2012.

**Resultados:**

Fueron hospitalizados 121 pacientes. Se realizaron pruebas para confirmar el diagnóstico (inmunofluorescencia directa, reacción en cadena de la polimerasa, cultivo) al 53,72%. El 23,15% (n = 28) fueron casos confirmados, todos menores de 10 meses, ninguno había recibido 3 dosis de la vacuna contra pertussis, el 96,43% (n = 27) de ellos fueron menores de 6 meses y 42,86% (n = 12) menores de 3 meses; un 10,71% (n = 3) ingresaron a unidad de cuidados intensivos, todos menores de 2 meses, uno de los cuales falleció. Los síntomas más frecuentes en los casos confirmados fueron tos (96,43%), rubicundez facial (96,43%), tos paroxística (92,86%) y cianosis asociada a la tos (78,57%); el contacto epidemiológico probable más frecuente fue la madre (17,86%) y la mayoría de casos se presentaron en verano (46,43%).

**Conclusión:**

La tos ferina es causa de morbimortalidad sobre todo en los menores de 6 meses de edad y en los no inmunizados o parcialmente inmunizados. Se deben mejorar las tasas de vacunación y fomentar la confirmación de casos para no contribuir al infradiagnóstico de esta enfermedad.

## INTRODUCTION

Whooping cough (WC), also called pertussis, *coqueluche*, or "100-day cough", is a highly contagious and immutable disease, with a secondary attack rate greater than 80%, and is caused by the bacterium *Bordetella pertussis*.^(1,2)^ The World Health Organization (WHO) estimated that there were 89,000 deaths due to this disease in 2008 and reported 143,661 cases worldwide in 2017.^(3-5)^ The highest rates of case reports continue to be in those younger than 1 year who are more likely to have complications and severe cases that can be fatal, especially for smaller infants.^(6-8)^ The most severe clinical presentation is malignant pertussis or severe pertussis, which can reach up to 75% mortality and is characterized by hyperleukocytosis, refractory hypoxemia, pulmonary hypertension, and respiratory failure.^(9-11)^

Vaccination is the main preventive strategy.^(12,13)^ Protection against severe cases with a single dose of the vaccine is 50 - 55.3%; with 3 doses, the immunity reaches 80-86%; and with 5 doses, immunity reaches 91%.^(12,14,15)^ This immunity drops by an average of 9.6% annually, leaving adolescents and adults exposed to contracting the disease and to becoming a source of infection.^(14-17)^ The WHO recommends compliance with 3 doses of WC vaccine in > 90% of infants.^(18)^ Younger infants who are not yet vaccinated or who have not yet completed the 3 doses are susceptible to becoming sick and to presenting as severe cases, necessitating strategies such as reinforcement in adolescents and the vaccination of pregnant women, among others, to protect these infants.^(19,20)^

In 2012, the WHO reported the largest number of WC cases in the last decade.^(5)^ In the same year, the United States reported the highest number of cases since 1955 (after the introduction of the vaccine), with 126.65/100,000,^(21)^ and in Peru, an epidemiological alert was declared, with a case rate (5.3/100,000) that was 20 times higher than the previous year.^(22,23)^ According to recent reports from Peru, another increase in cases was observed in 2017 (2.21/100,000), which was 3.6 times higher than that reported in 2016.^(24)^ We must take into account that the real incidence of WC may be higher due to underdiagnosis and to the lack of access to and performing of specific tests to confirm the diagnosis.^(25)^

WC continues to be a cause of child morbimortality worldwide and is a public health problem even in countries with high vaccination rates;^(7,26)^ therefore, it is necessary to understand its epidemiological characteristics to implement strategies to reduce the impact of this disease. In this study, we describe the epidemiological and clinical characteristics of patients younger than 2 years of age who were hospitalized with WC during 2012 in a tertiary care children's hospital in Lima, Peru.

## METHODS

This was a retrospective study of the clinical histories (HCLs) of patients under 2 years of age with a diagnosis of WC (International Classification of Diseases - ICD10 A37.0 and A37.9) who were hospitalized between January 1 and December 31, 2012, at the *Instituto Nacional de Salud del Niño* (INSN),^(27)^ a specialized tertiary-level pediatric hospital (category III-2) located in Lima, Peru. ICD10 A37.9 was included to review the cases for which the etiological agent was not identified. The list of HCLs was solicited from the INSN Statistics Office. In total, there were 123 cases; one case was excluded for being older than 2 years and another for being repeated. Of the 121 HCLs included in the study, 119 were reviewed, and information was obtained from the database of the intensive care unit (ICU) for the other 2 histories (belonging to two deceased patients).

The patients were divided into 3 groups: confirmed cases, probable cases, and suspected cases. For the confirmed and probable cases, the definitions of the Ministry of Health-Peru were used,^(28)^ which were valid during the study and remain valid at this time. The cases with clinical criteria for WC but lacking data on the time of illness, the epidemiological contact, or any performed confirmatory tests were classified as suspected cases (a definition also used by the Ministry of Health of Canada)^(13,29)^ for inclusion in the study to analyze their epidemiological variables.

Suspected cases had 1 of the following symptoms without other apparent cause: paroxysmal coughing of any duration, cough with inspiratory stridor, tussive vomiting, cough associated with apnea, or cyanosis.

Probable cases < 3 months of age had nonspecific upper respiratory tract infection associated with apnea and cyanosis, triggered by stimuli (e.g., feeding) and history of contact with a probable case of WC (person with cough ≥ 2 weeks and/or classic presentation of WC). Probable cases > 3 months of age had cough ≥ 2 weeks in duration with one or more of the following symptoms: paroxysmal coughing, inspiratory stridor, or tussive vomiting.

Confirmed cases were probable cases diagnosed using direct immunofluorescence (DIF) or polymerase chain reaction (PCR) and/or isolation of *B. pertussis* by culture; or probable cases with epidemiological link to a case confirmed by the laboratory during the period of transmissibility.

The immunization status was evaluated using the national vaccination schedule in effect at the time of the study as a reference, which was the 2011 Technical Standard for Vaccination.^(30)^ This document did not change until recently with respect to the vaccination of children against WC, as detailed in the recent vaccination schedule of 2018.^(31)^ The WC vaccine is administered along with the diphtheria and tetanus antigens (Tdap) included in the pentavalent vaccine (Tdap, hepatitis B and *Haemophilus influenzae* type B) to be administered at 2, 4, and 6 months, with the first reinforcement at 18 months and the second (and last) reinforcement at 4 years.^(30,31)^

Fisher's exact test was used to evaluate whether there were significant differences between the group that had mechanical ventilation (MV) versus non-MV patients. The statistical analysis was performed with the STATA version 15 package.

## RESULTS

**Figure 1:**
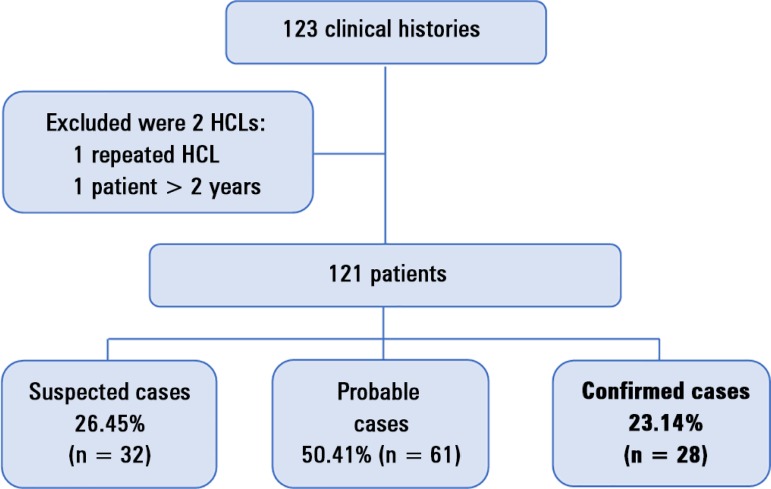
Patient flow chart. HCL - clinical histories.

There were 121 patients under 2 years of age hospitalized with a diagnosis of WC during 2012 at the INSN, and all were younger than 14 months (maximum age of 13 months). Two of the patients died (one case confirmed WC and one case suspected WC). Of the total cases, 23.14% (n = 28) were confirmed cases (Figure 1).

All confirmed cases (n = 28) were less than 10 months of age, with a mean age of 3.16 months (range 0.66 - 9.53); 96.43% (n = 27) were younger than 6 months, and 42.86% (n = 12) were younger than 3 months, and of these, one patient died (3.57%). The mean hospital stay was 12.46 days, with a standard deviation (SD) of 7.3. A predominance of these cases was observed in summer (January and February), with 46.43% (n = 13), followed by autumn (25%; n = 7), spring (25%; n = 7), and winter (3.57%; n = 1) (Table 1).

**Table 1:** Total cases, age, and hospital stay

	Suspected (N = 32)	Probable (N = 61)	Confirmed (N = 28)	Total (N = 121)
	**n (%)**	**n (%)**	**n (%)**	**n (%)**
Boys	21 (65.63)	29 (47.54)	13 (46.43)	63 (52.07)
Girls	11 (34.38)	32 (52.46)	15 (53.57)	58 (47.93)
	**Mean ± SD**	**Mean ± SD**	**Mean ± SD**	
Age (months)	2.37 ± 2.06	4.20 ± 3.00	3.16 ± 1.85	
Days of hospitalization	11.06 ± 10.04	9.90 ± 6.92	12.46 ± 7.3	

SD - standard deviation.

Probable epidemiological contact was recorded for 51.24% of all patients. For the confirmed cases, the mother was the probable epidemiological contact for 17.86% (n = 5), followed by siblings (14.29%; n = 4), aunts and uncles (14.29%; n = 4), cousins (10.71%; n = 3), and grandparents (3.57%; n = 1) who lived in the same home. Only two mothers were studied (one was mother of a 29-day-old infant and the other of a 4-month-old infant), and both had positive PCR samples for *B. pertussis.*

The clinical characteristics and complications are summarized in table 2. In the confirmed cases, the most frequent symptoms were cough (96.43%) and redness associated with cough (96.43%), followed by paroxysmal cough (92.86%) and cyanosis associated with cough (78.57%). Notably, 89.29% of confirmed cases also showed signs of obstructive pulmonary disease (OPD), and all those who presented with respiratory distress (35.71%) were younger than 5 months. During hospitalization, 96.43% (n = 27) of the confirmed cases required supplemental oxygen, of which 96.3% were younger than 6 months, and 72.92% had not received any dose of Tdap.

**Table 2:** Comparison of clinical characteristics, complications, and stay in the intensive care unit among the 3 groups of cases

	Suspected (N = 32)	Probable (N = 61)	Confirmed (N = 28)
	n (%)	n (%)	n (%)
Clinical characteristics			
Cough	31 (96.88)	61 (100)	27 (96.43)
Paroxysmal cough	23 (71.88)	52 (85.25)	26 (92.86)
Inspiratory stridor	2 (6.25)	6 (9.84)	2 (7.14)
Redness*	30 (93.75)	59 (96.72)	27 (96.43)
Cyanosis†	26 (81.25)	49 (80.33)	22 (78.57)
Tussive vomiting	9 (28.13)	27 (44.26)	13 (46.43)
Respiratory distress	6 (18.75)	11 (18.03)	10 (35.71)
Apnea	3 (9.38)	7 (11.48)	1 (3.57)
Seizure	1 (3.13)	1 (1.64)	0
OPD	25 (78.13)	44 (72.13)	25 (89.29)
Fever	11 (34.38)	24 (39.34)	8 (28.57)
Complications			
Pneumonia	8 (25.0)	22 (36.07)	9 (32.14)
Pneumothorax	0	1 (1.64)	1 (3.57)
Sepsis	0	0	2(7.14)
Septic shock	1 (3.13)	0	0
Cardiac arrest	2 (6.25)	0	1 (3.57)
Deceased	1 (3.13)	0	1 (3.57)
Patients in ICU	4 (12.5)	5 (8.19)	3 (10.7)
Hospitalized days (mean, range)	26 (2 - 43)	22.8 (12 - 37)	23.66 (18 - 33)
Days in ICU (mean, range)	16 (1 - 32)	13.4 (6 - 25)	8.3 (4 - 12)
Patients on MV	3 (75)‡	4 (80)‡	2 (66.6)‡
Days in MV (mean, range)	11 (2 - 20)	13.5 (4 - 23)	9.5 (9 - 10)

OPD - obstructive pulmonary disease; ICU - intensive care unit; MV - mechanical ventilation.

* Redness associated with cough;

† Cyanosis associated with cough.

‡ Percentage of patients on mechanical ventilation compared to the total number of patients in the intensive care unit.

None of the confirmed cases (n = 28) had received 3 doses of WC vaccine (Tdap). In total, 75% (n = 21) did not have any dose, 14.29% had only 1 dose, and 3.57% (n = 1) had 2 doses; the vaccination status of one 5-month-old patient was not known. Regarding vaccination compliance according to age, children under 2 months (35.71%) had not received the first dose because they had not yet begun primary vaccination. Of the patients between 2 and 4 months (n = 10), 3 of them (10.71%) had received 1 dose of Tdap. Of the patients between 4 to 6 months (n = 6), 2 had received 2 doses, and the only patient older than 6 months (9 months) had not received any dose of vaccine (Table 3).

**Table 3:** Confirmed cases and doses of diphtheria, pertussis, and tetanus vaccine according to age

	Total confirmed cases (n: 28 = 100%)	
	Number of cases/number of cases total per age	Cases with vaccination according to age %
Under 2 months without Tdap dose	10/10	35.71
From 2 to 4 months with 1 Tdap dose	3/10	10.71
From 4 to 6 months with 2 Tdap doses	2/7	7.14
Older than 6 months with 3 Tdap doses	0/1	0
Unknown	1	3.57

Tdap - vaccine against diphtheria, pertussis, and tetanus.

Diagnostic testing: PCR, DIF, and culturing for *B. pertussis* were performed for 53.72% (n = 64) of the patients. PCR was performed for 25.62% (n = 31), with 26 positive results, constituting 92.86% of confirmed cases. The culture of pharyngeal secretion was performed in one patient, with a positive result. DIF was carried out in 32.23% (n = 39) of cases, with one positive and 38 negative results, of which 6 cases were confirmed by PCR, thus finding 15.79% false negatives.

The serological IgM and IgG testing for *B. pertussis* was qualitative. IgM testing was performed in 49.59% (n = 60) of cases, with 11 positive results, 4 of which were confirmed by PCR. IgG testing was performed in 2 patients (1.65%), both with negative results, and one of them was confirmed as a positive case by PCR. These tests were not considered as confirmatory tests for WC in this study because they are not confirmatory criteria according to the Ministry of Health.

Indirect immunofluorescence (IIF) and DIF tests were performed for virus detection in 4.96% (n = 6) of cases, being negative in 5 of them and positive for metapneumovirus in a patient who also had a positive PCR result for *B. pertussis*.

The presence of leukocytosis (> 11,000/mm^3^) with lymphocytosis (> 70%) in the complete blood count (CBC) on admission was found in 35.71% of confirmed cases. No confirmed cases of hyperleukocytosis (leukocytes > 100,000/mm^3^) were found. A similar percentage of cases with leukocytosis plus lymphocytosis was found in the suspected cases (34.37%) and in the probable cases (24.59%). Thrombocytosis (platelets > 600,000/mm^3^) was observed in 60.71% of the confirmed cases, in 57.38% of probable cases, and in 62.5% of suspected cases. The only patient with hyperleukocytosis was a probable case who was 6 months old and had not received any dose of Tdap. This patient had fever, pneumonia, and paroxysmal cough and was admitted due to suspected lymphoproliferative syndrome, with leukocytes = 107,000/mm^3^. The patient received macrolide antibiotic treatment and was discharged after 8 days of hospitalization upon normalization of the blood count.

All patients were treated with macrolides when WC was suspected, and azithromycin was the most indicated (85.12%, n = 103). In confirmed cases, 92.86% received azithromycin, 3.57% received erythromycin, and none received clarithromycin; the macrolide given to one patient was not known.

Of the confirmed cases, 28.57% received non-macrolide antibiotic treatment, 89.29% received β2 agonist bronchodilators (salbutamol), and 7.14% received nebulization with 3% hypertonic serum. All patients receiving salbutamol were diagnosed with OPD. Intravenous systemic corticosteroids (hydrocortisone, methylprednisolone, dexamethasone) and oral corticosteroids (prednisone, prednisolone) were indicated in 42.86% (n = 12); 7.4% (n = 2) received inhaled corticosteroids (fluticasone, beclomethasone, budesonide), and oxolamine and dextromethorphan were indicated in 17.86% (n = 5).

The ICU and MV durations of the patients requiring intensive care were similar in the 3 groups of cases (Table 2). Overall, 9.92% (n = 12) were younger than 5 months, and 83.33% (n = 10) were younger than 2 months without having received any WC vaccine, of which 2 patients died. Of these, one case was confirmed by PCR (case no. 3, Table 4), and one was a suspected case. The latter was 1 month and 16 days old and was hospitalized with a diagnosis of WC and pneumonia syndrome. This patient was admitted to the ICU at 24 hours due to respiratory distress and was placed in MV. Septic shock followed, and the patient died 24 hours after admission to the ICU; no confirmatory tests were performed for WC. Of all patients who required MV, the majority (88.89%; p = 0.002) presented some complications of WC (apnea, seizures, pneumonia, pneumothorax, myocarditis, sepsis, and/or septic shock).

**Table 4:** Confirmed cases in the intensive care unit

N	Age	Sex	Tdap vaccine	*Leukocytes (% lymphocytes)	Pneumonia	Sepsis	Pneumothorax	Myocarditis	MV/days	Outcome (hospitalization days)
1	1 month, 18 days	F	none	9800 (68)	No	No	No	No	No	High (18 days)
2	1 month, 15 days	F	none	8370 (72)	Yes	Yes	Yes	No	Yes/ 10 days	High (33 days)
3	1 month, 11 days	M	none	UN	No	Yes	No	Yes	Yes/ 09 days	Deceased (20 days)

Tdap - diphtheria, pertussis, and tetanus vaccine; MV - mechanical ventilation. UN - unknown; F - female; M - male.

* Leukocyte and lymphocyte count at hospital admission.

Of the confirmed cases, 39.29% (n = 11) had complications, all were younger than 6 months, and 8 of them (72.73%) were unvaccinated. A total of 10.71% (n = 3) of confirmed cases were admitted to the ICU, and all had respiratory distress, were under 2 months of age, and had not received the first dose of Tdap. The characteristics of the confirmed cases in the ICU are summarized in table 4.

Only one patient with a serious diagnosis of WC was found. This was a 1-month-old infant who was hospitalized with a diagnosis of syndrome WC and pneumonia plus atelectasis. The CBC at admission was as follows: leukocytes 9,400/mm^3^, lymphocytes 47%, and platelets 673,000/mm^3^. At 48 hours, the patient was admitted to the ICU due to respiratory distress and received intravenous Ig (5 days) for the proposed diagnosis of severe WC. The DIF and IgM analyses for *B. pertussis* were negative, no PCR was performed, no diagnosis of pulmonary hypertension was found, and the patient was discharged 25 days after hospitalization.

## DISCUSSION

Whooping cough is a worldwide endemic disease, and despite vaccination, cases continue to occur every 3 to 5 years.^(3)^ The largest peak of WC in recent years in Peru was in 2012, when 20 deaths were reported.^(22)^ This year had the highest incidence for other countries in the Americas, such as the United States, Canada, Chile, Mexico, and (in 2011) Argentina.^(5)^ In this study, we found 121 patients under 2 years of age with a diagnosis of WC admitted during 2012 in a tertiary hospital in Lima, Peru. Of these, 23.14% (n = 28) were confirmed cases.

Whooping cough is a disease that is subject to epidemiological surveillance, which is why there is little concern about the search for a confirmation of diagnosis and few epidemiological records in this study. One of the difficulties in performing PCR in the studied hospital was the limited accessibility to this type of test and to the DIF. These tests are not performed in the studied hospital but at another institution, the INS (National Institute of Health), where the sample is taken after having been evaluated by the epidemiology staff of the hospital. In addition, the supplies for performing PCR are not always available; therefore, DIF is often performed instead. However, the use of DIF has been discouraged as a WC confirmatory method by the WHO,^(3)^ the Centers for Disease Control and Prevention (CDC),^(32)^ and the Global Pertussis Initiative^(13)^ due to its low sensitivity and specificity. In our study, DIF had a 15.79% false negative rate.

The lack of a consensus regarding the definitions of cases and confirmatory methods hinders the overall analysis of this disease. In a meta-analysis of WC in Latin America and the Caribbean,^(33)^ the difficulty of comparing the incidence of WC among countries is mentioned, where some use WHO definitions and others use national definitions (as in our study). We believe that it is important to standardize these definitions to compare the incidence among countries, to better understand the true burden of this disease, to avoid underdiagnosis, and to implement epidemiologically appropriate preventive strategies.

In the 3 groups of cases, similar results were found with respect to the variables analyzed, so it is possible that some or all of the suspected and probable cases were true cases of WC that were not identified due to lack of confirmatory study. However, because we cannot know this for sure, we will limit our discussion to the results of the confirmed cases.

In Latin America and the Caribbean between 2006 and 2015, the countries with the highest rates of WC cases in children under 1 year were Costa Rica (> 45/100,000), Chile (30.71/100,000), Uruguay (24.82/100,000), and Argentina (13.88/100,000).^(34)^ Spain, a country that has a high vaccination rate,^(35)^ reported an increase in incidence in 2015, especially in children under 1 year of age (457.2/100,000).^(36)^ This age group continues to have the highest reported rates, and of these, 50-90% are hospitalized.^(7)^ This finding coincides with the findings of our study, where 100% of confirmed hospitalized cases were less than 10 months of age, and of these patients, 96.43% were younger than 6 months. Other authors, such as Kusznierz et al.^(37)^ in Santa Fé, Argentina, have found that all hospitalized patients were under 1 year of age, of whom 94.2% were younger than 6 months and 67.6% were younger than 2 months. In Barcelona, Spain, Urima Tuma et al.^(38)^ found that 80.3% of hospitalized cases were under 6 months of age. In the state of Paraná, Brazil, Torres et al.^(39)^ concluded that children under 1 year were the age group most affected by WC (67.5%), especially those younger than 2 months. These studies show that the highest prevalence of hospitalization occurs in children under 1 year of age and especially in those younger than 6 months.

In Latin America, coverage of the 3 doses of Tdap (Tdap3) was lower in the two lowest income quintiles between 2000 and 2015.^(34)^ In Peru, the coverage of Tdap3 has decreased in recent years, from 90% in 2015 to 89% in 2016 and 83% in 2017.^(40)^ These data are of concern because immunization is the main preventive strategy against severe cases of WC. The lethality of this disease as described by Folaranmi et al.^(33)^ in a meta-analysis of WC in Latin America and the Caribbean was 3.9%. The WHO states that the lethality reaches 4% in developing countries.^(3)^ Kusznierz et al.^(37)^ found a lethality of 4.9%, and all were younger than 2 months and had not received the first vaccine dose. In Sweden, Carlsson et al.^(41)^ found a lethality of 0.65%, and all were younger than 6 months; Aristimuño et al.^(42)^ in Gipuzkoa, Spain, found a lethality of 1.85%, and all were less than 2 months of age. In our study, we found a lethality of 3.57%, and these were infants younger than 2 months without any dose of WC vaccine. The lethality of WC varies among countries due to factors such as vaccination and epidemiological surveillance. However, the above studies show that the smallest and unimmunized infants are those with a higher risk of mortality.

The characteristics found in patients admitted to the ICU (age, mean number of days of hospitalization, ICU stay, and MV) were similar to those found by other studies. Kusznierz et al.^(37)^ described 23.1% of patients admitted to the ICU, with an average stay of 7 days, all less than 2 months of age and without any dose of the vaccine. Palvo et al.^(43)^ found in a tertiary hospital in Brazil that of the 82.35% of cases admitted to the ICU who were younger than 3 months, 76.5% were unvaccinated, and those who needed MV remained there for a mean of 7 days. In a multicenter study in the United States, Berger et al.^(44)^ found that 83% of ICU patients were under 3 months of age, 74% were unvaccinated, and those who were on MV remained there for an average of 8 days, with an average ICU stay of 11.8 days. The results of these studies agree that children under 3 months of age and those not immunized have a higher risk of severe WC, reinforcing the importance of implementing preventive strategies to protect this age group.

Several studies conclude that the main epidemiological contact is household contact. In the United States, Skoff et al.^(45)^ first found siblings (35.5%), followed by mothers (20.6%) and fathers (10%), as the main epidemiological contact. In Chile, Perret et al.^(46)^ found mainly mothers and fathers (40%) to be the primary contact, and Uriona Tuma et al.^(38)^ concluded that mothers were the main source of infection (21%). In our study, the recorded epidemiological contacts were all from the household, and these were mainly mothers. This evidence supports the strategy of vaccinating pregnant women to protect infants and toddlers, as recommended by the Global Pertussis Initiative,^(19)^ the CDC,^(20)^ and the WHO^(3)^ and implemented in countries such as the United States since 2011,^(47)^ Argentina^(48)^ and United Kingdom^(49)^ since 2012, Spain since 2015,^(36)^ and recently in Peru since August 2018.^(31)^ Likewise, this evidence supports the strategy of applying reinforcement vaccinations in children and adolescents to reduce the potential source of contagion for children.

## CONCLUSION

In the present study, whooping cough was found to be a cause of morbidity and mortality in children less than 1 year of age, especially in children under 6 months, and in those who were not immunized. It was not possible to conclude whether there would be a higher incidence of whooping cough due to the lack of confirmatory tests in suspected and probable cases. Regardless, with the confirmed cases that were found, important epidemiological information was obtained that reinforces and supports the findings of other studies. Our findings also support the suggestions to improve our vaccination rates, continue implementing new strategies for the prevention of this disease, and improve epidemiological surveillance.
